# Real‐world evidence of tirbanibulin in the treatment of actinic keratosis in Germany – prospective, multicenter, non‐interventional KLIR study

**DOI:** 10.1111/ddg.15876

**Published:** 2025-09-25

**Authors:** Markus V. Heppt, Ina Hadshiew, Afra Kempf, Antje Melzer, Carola Berking

**Affiliations:** ^1^ Department of Dermatology University Hospital Erlangen Friedrich‐Alexander University Erlangen‐Nürnberg Erlangen Germany; ^2^ Comprehensive Cancer Center Erlangen‐EMN Erlangen Germany; ^3^ Bavarian Cancer Research Center (BZKF) Erlangen Germany; ^4^ MVZ Derma Cologne Cologne Germany; ^5^ Almirall Hermal Reinbek Germany

**Keywords:** Actinic keratosis, non‐interventional study, patient‐reported outcomes, real‐world evidence, tirbanibulin

## Abstract

**Background:**

Tirbanibulin is an approved topical treatment for actinic keratosis (AK). This non‐interventional study investigated effectiveness and safety of tirbanibulin for AK.

**Patients and Methods:**

The prospective KLIR‐study was conducted at 58 German dermatology centers (02/2022–09/2023). Patients received tirbanibulin once daily for 5 consecutive days. Lesion count reduction, clearance rates, safety, and treatment satisfaction were assessed at day 57 (visit 3) after treatment. Visits on days 0, 8–29, and 240 allowed for the evaluation of baseline data, local skin reaction (LSR) peaks, and lesion recurrence, respectively.

**Results:**

In total, 545 AK patients were included in the study. The mean number of AK lesions decreased from 5.9 (baseline) to 1.9 at visit 3, representing a 70% lesion count reduction (*p* < 0.0001). Clearance rates at visit 3 were 37.4% (complete clearance) and 55.0% (partial clearance ≥ 75% reduction). Among patients with complete clearance at visit 3, 21.9% experienced recurrent AK lesions at visit 4. The majority of LSRs were mild or moderate. Erythema (97.6%) was the most common LSR. Overall, 91.5% of patients rated their AK lesions as improved and 99% would consider future tirbanibulin treatment (“definitely”, “certainly”, “probably”, “maybe”).

**Conclusions:**

Tirbanibulin showed high lesion clearance and a high level of acceptance in real‐life conditions.

## INTRODUCTION

Actinic keratosis (AK) is a common precursor lesion of cutaneous squamous cell carcinoma (SCC) that predominantly affects sun‐damaged skin.^1^, ^2^ In Europe, the prevalence of AK is approximately 18%.^3^ Given the potential for progression to invasive cancer, early detection and treatment of AK is critical.

AKs are conventionally classified clinically according to Olsen (grade I–III) and histologically (keratinocyte intraepidermal neoplasia [KIN] I–III) using three‐tiered systems.^4^, ^5^ The categorization of KIN I is determined by the extent of atypia affecting the basal keratinocytes limited to the lower third of the epidermis. In contrast, KIN II is characterized by atypia affecting the lower two thirds of the epidermis, while KIN III is defined by the presence of atypical cells in all layers of the epidermis.^5^, ^6^, ^7^ It has been assumed that the progression from AK to SCC occurs via a sequential progression of the three KIN stages. Consequently, KIN I lesions have been considered to pose a relatively low risk of progression to SCC. Nevertheless, recent evidence indicates that SCC can already develop from low‐grade AKs as studies have identified KIN I lesions as the most common precursor of cutaneous SCC.^6^, ^8^ Although many AKs will either resolve or remain at the same stage, the progression potential of the individual AK is not predictable. Therefore, monitoring and treatment of all AKs is uniformly recommended in national and international guidelines, regardless of their grade.^9^, ^10^

There are many therapeutic options available for the treatment of AK, ranging from physical treatments such as cryotherapy and photodynamic therapy, to ablative regimens and topical therapies.^1^, ^11^ Tirbanibulin 1% (Klisyri^®^), a topical ointment for the treatment of AK, was approved by the *European Medicines Agency* in July 2021 and by the *US Food and Drug Administration* in December 2020.^12^, ^13^ Tirbanibulin is indicated for the field treatment of non‐hyperkeratotic, non‐hypertrophic AK (Olsen grade I) of the face and scalp in adults. Unlike other topical therapies, tirbanibulin requires a short treatment protocol of a once‐daily application for five consecutive days. Its tolerability and efficacy in reducing AK has been demonstrated in a phase I (NCT02337205), a phase IIa study (NCT02838628) and two double‐blind, placebo‐controlled, randomized phase III studies (NCT03285477 and NCT03285490). These studies showed that tirbanibulin mainly induces mild and transient treatment‐related adverse events.^14^ In addition, tirbanibulin applied once daily for 5 consecutive days on the face or scalp over a field up to 25 cm^2^ was able to clear lesions completely after 2 months in 44% and 54% of the patients in the first and second phase III study, respectively.^3^ The mean number of lesions on day 57 was reduced by 76% (trial 1) and 82% (trial 2), respectively, in the tirbanibulin group.^3^, ^14^ As two recent US studies reported safety and tolerability of tirbanibulin applied to a field of approximately 100 cm^2^, ^15^, ^16^, the FDA approved a *supplemental New Drug Application* (sNDA) for tirbanibulin applied to an area of 100 cm^2^.

These clinical trials assessed efficacy and safety in a specific patient population defined by strict inclusion criteria. Therefore, the value of these trials in predicting efficacy in a real‐world setting is limited. A more accurate presentation of the effectiveness and convenience of treatments can be obtained from non‐interventional studies (NIS) providing real‐world evidence. Two rather small tirbanibulin‐related real‐world studies (33 and 38 patients) and one retrospective study with 250 patients have already demonstrated the effectiveness and safety of tirbanibulin for the treatment of AK.^17^, ^18^, ^19^ In addition, a tirbanibulin studie involving 290 patients in a real‐world community practice in the US revealed successful treatment outcomes, improved quality of life and resulted in high levels of treatment satisfaction.^20^

Given the limitations of the patient cohort and the lack of real‐world data in German AK patients, the aim of this NIS was to collect and evaluate real‐world treatment data on effectiveness and safety of tirbanibulin as well as patient‐reported outcomes (PROs) in a large cohort from predominantly outpatient settings in Germany.

## PATIENTS AND METHODS

### Study design, setting

KLIR (Klisyri^®^ in Real World Treatment) was a prospective, open‐label, multicenter NIS conducted in 58 German dermatology centers between February 2022 and September 2023. This NIS was carried out in accordance with the joint recommendations of the *Federal Institute for Drugs and Medical Devices* (BfArM) and the *Paul Ehrlich Institute* (PEI) for the planning, conduct and analysis of observational studies (§ 4(23) and § 67(6) of the *German Medicines Act*). Prior to the start of the study, all relevant documents were reviewed by the ethics committee of Friedrich‐Alexander‐Universität Erlangen‐Nürnberg (No. 21‐444‐NIS; approval granted on December 15, 2021). The data collection process was performed in accordance with the current version of the *Declaration of Helsinki* as well as the relevant guidelines and recommendations for good epidemiological practice. Prescription of tirbanibulin 1% ointment, visit schedule and data documentation during this NIS strictly followed routine clinical practice in Germany.

The response assessment period comprised three visits: visit 1 at baseline, visit 2 scheduled between days 8–29 (optional), and visit 3 scheduled for day 57. Thus, the general study duration until the end of the response assessment period should comprise approximately 8 weeks (depending on the actual timing of patients’ appointments), in accordance with the treatment recommendations and durations outlined in the Summary of Product Characteristics (SmPC). This was followed by an optional long‐term follow‐up of approximately 6 months (visit 4, scheduled on day 240), resulting in a total documentation period of approximately 8 months per patient.

### Participants

Adult (≥ 18 years) patients with AK who were eligible for treatment with tirbanibulin 1% ointment according to the approved SmPC and who signed an informed consent form were included in the study.

### Procedure and assessment

**FIGURE 1:**
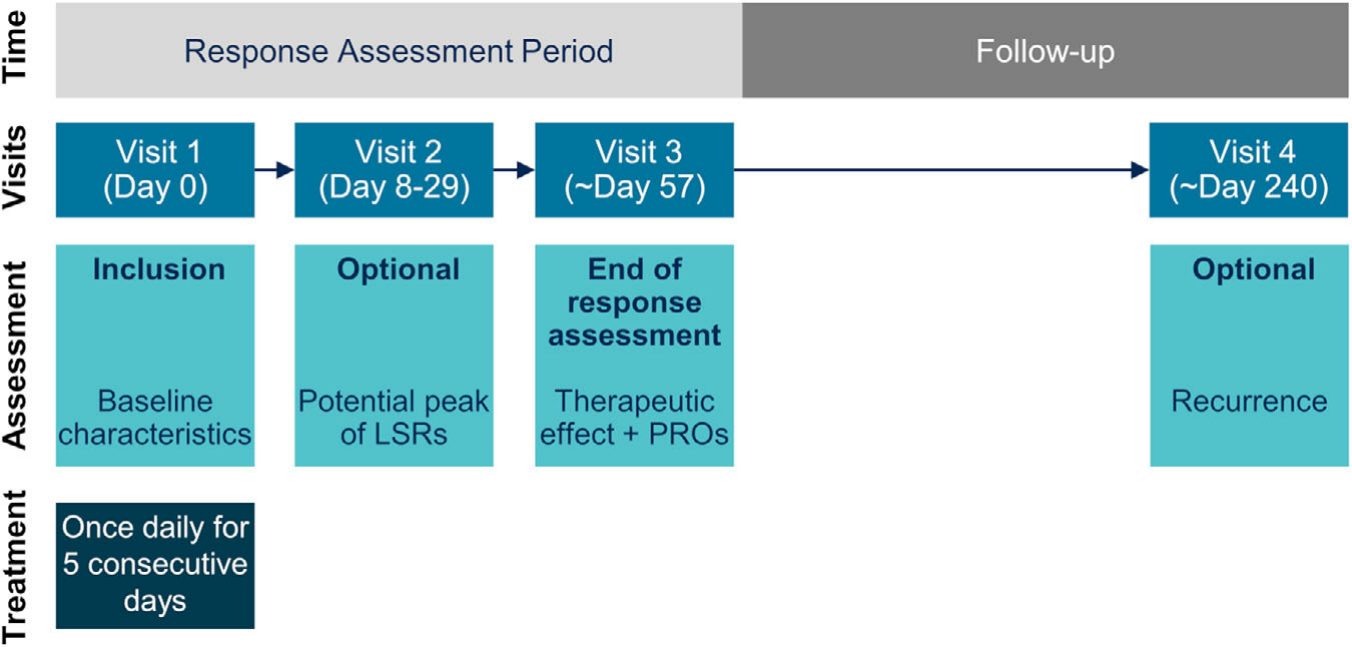
Treatment and visit schedule of KLIR.

The treatment and visit schedule of the KLIR study is shown in Figure 1. Study enrollment was performed at the initial visit (visit 1). Baseline data included the Fitzpatrick skin type, number and location of lesions in the treatment area (5 x 5 cm), and prior AK treatments within the previous 12 months (according to the case report form). Tirbanibulin 10 mg/g (ointment 1%) should be applied based on the approved SmPC. One primary objective was the safety and tolerability of tirbanibulin as documented by adverse events (AEs), including adverse drug reactions (ADRs) and adverse events of special interest (AESIs) at the treatment site in all patients who received at least one dose of tirbanibulin. AESIs were defined as the development of skin tumors in the treatment area. Local skin reactions (LSRs) were documented separately from above‐mentioned safety events. AEs and LSRs were recorded continuously throughout the study. In addition, a potential peak in LSRs was assessed during the optional visit 2. Therapeutic effectiveness, as measured by lesion reduction and clearance rates (complete clearance and partial clearance [≥ 75% reduction]), was assessed at visit 3 compared to baseline. In addition, participating physicians, patients and their partners reported their treatment satisfaction at visit 3 (additional objectives). Physicians’ satisfaction was determined using a 4‐point scale (very satisfied, satisfied, dissatisfied, very dissatisfied). To capture PROs, standardized and routinely used questionnaires, including the *Patient Global Improvement Index* (PGII)^21^, a cosmetic assessment scale and a future treatment preference questionnaire were used. Using the PGII, patients rated their disease status compared to baseline on a 7‐point scale (completely improved [healed], significantly improved, moderately improved, slightly improved, no change, slightly worse, significantly worse). Patients and their partners rated the cosmetic outcome on a 4‐point scale (much improved, somewhat improved, no change, somewhat worsened). Patient's future treatment preference referred to the willingness to repeat therapy with tirbanibulin and was assessed using the 5‐point *Patient's Future Treatment Preference Scale* (definitely, certainly, probably, maybe, not at all). Adherence was assessed by documenting the number of days tirbanibulin was applied. Long‐term follow‐up was performed at visit 4 to assess safety and recurrence or development of new lesions in the treatment area in patients who achieved complete clearance at visit 3 (additional objective). Data were collected for each patient using paper‐based case report forms and PRO forms.

### Statistical analysis

Statistical analyses were performed by using descriptive methods. An observed‐case approach was employed to tabulate data by visit. Continuous variables are described by the number of non‐missing values, number of missing values, arithmetic mean, standard deviation, median, minimum value, and maximum value. Categorical variables are presented as absolute frequency and the relative percentage for each observed modality. Clearance rates were calculated as percentages by visit with 95% Clopper‐Pearson exact confidence intervals (CI). Unless otherwise stated, two‐sided 95% CIs were derived. Similarly, p‐values were two‐sided and interpreted using a 5% significance level. A subgroup analysis of clearance rates for patients with visit 3 between days 47 and 67 was performed.

As this was a NIS, no formal sample size calculation was required. In general, missing values were not imputed and data were analyzed as they appeared in the clinical database, except for incomplete stop dates for diagnosis of AK, previous AK treatment, and concomitant medication. If a day was missing, the last day of the respective month was imputed. Otherwise, no imputation was performed, and the date was treated as missing.

The statistical analysis of this study was performed using the SAS^®^ software version 9.4 (SAS Institute Inc., Cary, NC, USA).

## RESULTS

### Patient disposition, demographics and baseline characteristics

A total of 545 patients were enrolled in the study. Of these, two were not treated and one patient did not meet the eligibility criteria. Thus, the safety and efficacy population comprised 543 and 542 patients, respectively. The second optional visit to assess LSRs was attended by 516 patients after a mean duration of 21. 7 days (± 20.7 days), while 507 patients were reported for response assessment after a mean duration of 72.4 days (±25.3 days) (visit 3). A total of 437 patients were assessed at the optional visit 4 to evaluate AK recurrence.

**FIGURE 2:**
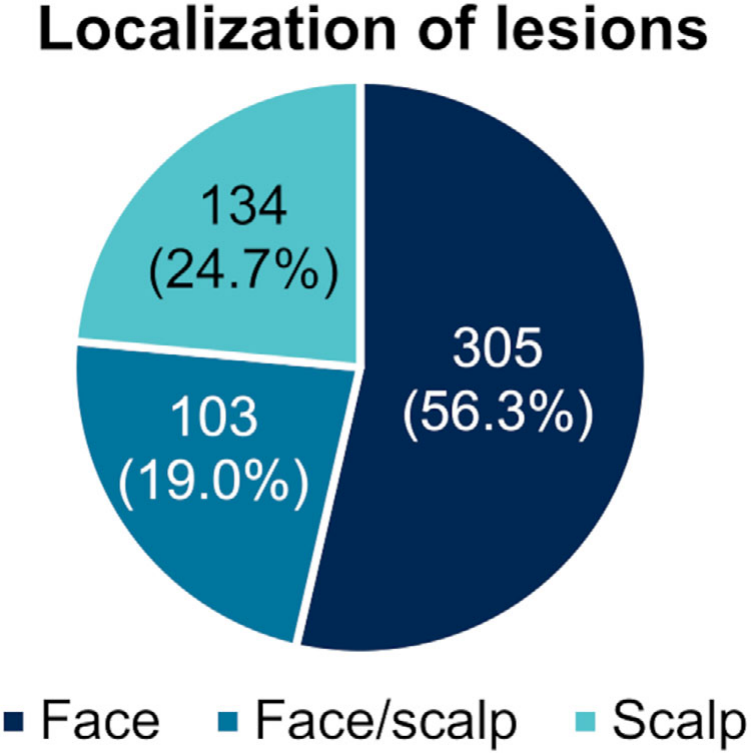
Localization of AK lesions at baseline (n = 542).

The median age of the patients at baseline was 74 years (range 40–99), 67.8% were male (Table 1), and the majority presented with Fitzpatrick skin types 2 (52.1%) and 3 (22.7%). A mean number of 5.9 (± 4.6) lesions were observed in the treatment area, predominantly on the face (n = 305; 56.3%) and less commonly on the scalp (n = 103; 24.7%) and on both areas (n = 130; 19.0%) (Figure 2). More than half of the patients had received at least one previous treatment for AK in the 12 months prior to the study (n = 298; 54.9%), most commonly applied were topical medical treatments (n = 158; 29.1%), followed by cryotherapy (n = 129; 23.8%) (Table 1). Patients with immunosuppression were not excluded from the study and the study included twelve patients receiving immunosuppressive drugs and two patients with hematologic malignancies (acute myeloid leukemia [AML] and chronic lymphatic leukemia [CLL]). The median treatment duration of tirbanibulin was 5 days (range: 1–8), and 90.3% of patients (437 out of 484 patients [observed cases]) administered tirbanibulin five times.

**TABLE 1:** Patient demographics and baseline characteristics.

Parameter	n = 543
***Demographic data***	
Age (years), median (range)	74.0 (40–99)
Male, n (%)	368 (67.8)
Number of clinically assessed lesions, mean (SD)	5.9 (± 4.6)
***Previous AK therapy, n (%)***	
Yes	298 (54.9)
No	239 (44.0)
Previous medical treatment^*^	
Chemical peeling	32 (5.9)
Cryotherapy	129 (23.8)
Laser ablation	6 (1.1)
Photodynamic therapy	45 (8.3)
Surgical procedure	7 (1.3)
Systemic medical therapy	2 (0.4)
Topical medical treatment	158 (29.1)
Other therapy	3 (0.6)

*Abbr*.: n, number of patients in analysis population; n, number of patients among n; (%), percentage among n; SD, standard deviation; Missing, number of missing observations among n

* Multiple answers were possible.

### Effectiveness

**FIGURE 3:**
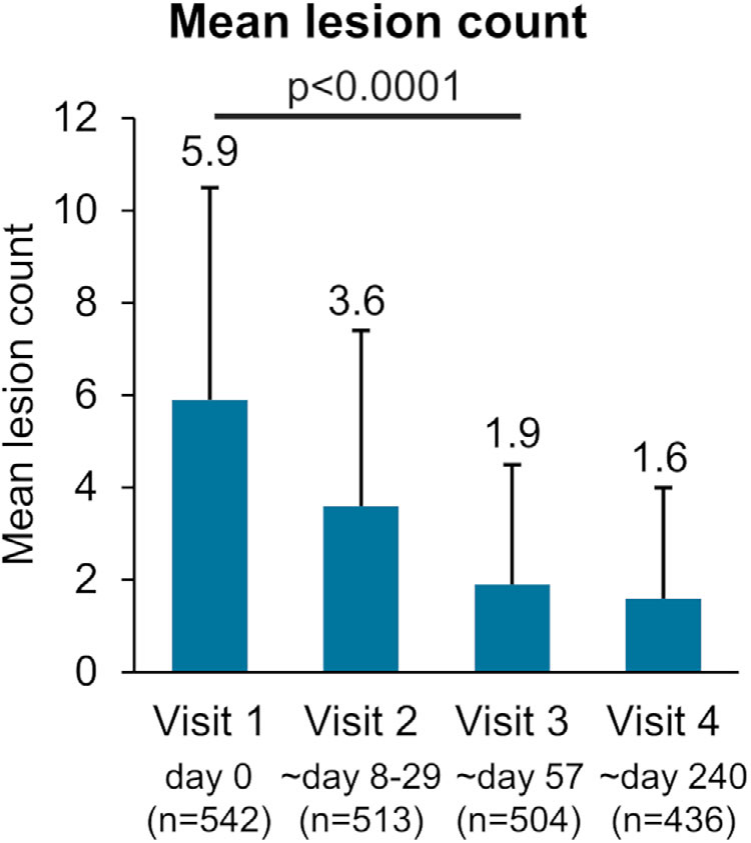
Mean lesion count and SD at baseline (visit 1 [n = 542]), at visit 2 (n = 513), at visit 3 (n = 504), and at visit 4 (n = 436).

The mean number of lesions decreased from 5.9 (± 4.6) at baseline to 1.9 (± 2.6) at the end of the response assessment period (visit 3), resulting in a mean reduction of 4.1 lesions (95% CI –4.5 to –3.8). At visit 4, the mean number of lesions was 1.6 (± 2.4) with a mean reduction of 4.5 lesions from baseline (Figure 3). Overall, there was a significant reduction of 70% (*p* < 0.0001) and a reduction of 73% in the total lesion counts from baseline to a mean of 72.4 days and 240 days, respectively.

Clearance rates were determined at the end of the response assessment period (scheduled on day 57). However, due to the non‐interventional design of the study, no fixed visit schedule was available. As visit 3 was reported between day 26 and day 248, clearance rates were calculated separately for the overall group (n = 505) and for a subgroup (n = 277) that included only patients with visit 3 between day 47 and day 67, according to the SmPC (day 57 ±10 days). For the overall group, 189 out of 505 patients (37.4%; 95% CI 33.2–41.8) achieved complete clearance of lesions compared to baseline. Partial clearance was observed in 278 patients (55.0%; 95% CI 51.6–60.5). In the subgroup of patients with a visit 3 between day 47 and day 67, 127 patients (45.8%; 95% CI 39.9–51.9) and 178 patients (64.3%; 95% CI 58.3–69.9) out of 277 patients achieved complete and partial lesion clearance, respectively.

For patients with complete clearance at visit 3, the recurrence rate of AK lesions in the treated area during follow‐up (visit 4) was 21.9% (34 out of 155).

### Safety and tolerability

A total of 23 AEs were reported in 13 patients (2.4%) (Table 2), including three serious adverse events (n = 2 [0.4%]), none of which were considered treatment‐related. One patient was hospitalized for fever, and another patient was hospitalized once for cardiac catheterization and once for pacemaker replacement. Notably, none of the AEs led to dose changes or discontinuation of study or treatment with tirbanibulin. Importantly, no AESIs were observed (Table 2). About half of the AEs were classified as ADRs (12 events in 4 patients). The most frequently documented ADRs were related to skin and subcutaneous tissue disorders, observed in three patients (0.6%) (Table 2). These included erythema, atopic dermatitis, and skin exfoliation. Except for one case of moderate erythema, all events in this category were of a mild severity.

**TABLE 2:** Adverse events (multiple answers possible).

	n = 543	
n (%)	n (%)	Events
AE total	13 (2.4)	23
Serious adverse event (SAE)	2 (0.4)	3
Premature discontinuation of study due to AE	0 (0.0)	0
Premature discontinuation of treatment due to AE	0 (0.0)	0
Adverse drug reactions (ADR)	4 (0.7)	12
Adverse event of special interest (AESI)	0 (0.0)	0
***Adverse drug reactions*** ^*^		
Eye disorders	2 (0.4)	2
Allergic conjunctivitis	1 (0.2)	1
Eye irritation	1 (0.2)	1
Gastrointestinal disorders	1 (0.2)	1
Stomatitis	1 (0.2)	1
General disorders and administration site conditions	2 (0.4)	2
Influenza like illness	1 (0.2)	1
Swelling	1 (0.2)	1
Respiratory, thoracic and mediastinal disorders	1 (0.2)	1
Nasal dryness	1 (0.2)	1
Skin and subcutaneous tissue disorders	3 (0.6)	6
Atopic dermatitis	1 (0.2)	1
Erythema	2 (0.4)	3
Skin exfoliation	1 (0.2)	2

*Abbr*.: n, number of patients in analysis population; n (%), number of patients and percentage among n; [Events], number of individual events which occurred among the n patients

* Multiple answers were possible.

LSRs were documented separately from above‐mentioned safety events. In 538 of 543 patients (99.1%), at least one LSR was documented (total across all visits), but none was considered serious. The most common LSRs were “erythema” (97.6%) and “flaking/scaling” (90.0%), followed by “crusting” (63.6%), “swelling” (20.8%), “erosion/ulcers” (17.1%) and “vesicles/pustules” (8.9%). Most LSRs were of “mild” (65.4%) and “moderate” (29.5%) severity.

### Physicians’ satisfaction and patient‐reported outcomes (PROs)

**FIGURE 4:**
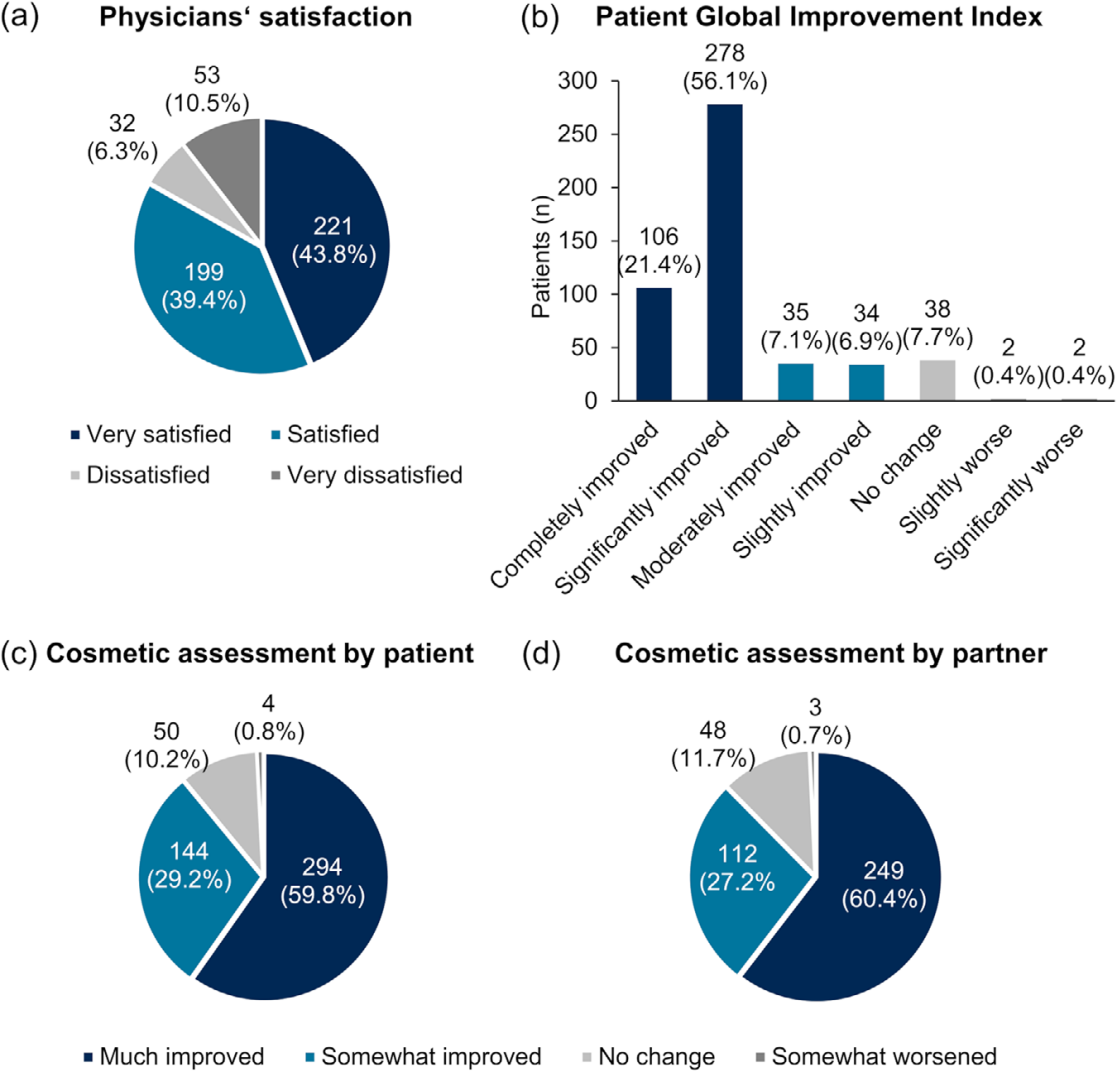
(a) Satisfaction of participating physicians with treatment outcome (n = 505), (b) satisfaction of patients with improvement in disease pattern and symptoms (n = 495), (c) satisfaction with cosmetic outcome as rated by the patients (n = 492), and (d) satisfaction with cosmetic outcome as rated by their partners (n = 412) at visit 3 (approximately day 57).

Participating physicians were mostly satisfied with the treatment outcome in 83.2% (420 out of 505) of patients (“very satisfied”, 43.8%, and “satisfied”, 39.4%) at the end of the response assessment period (Figure 4a).

A total of 91.5% of patients (453 out of 495 patients) rated their AK lesions and associated symptoms as improved at visit 3. Compared to baseline, 77.5% of patients rated their symptoms as “completely improved” and “significantly improved”, 14.0% as “moderately improved” and “slightly improved”. “No change” or worsening of the symptoms compared to prior treatment was reported by 7.7% and 0.8% of patients, respectively (Figure 4b). Accordingly, at the end of the response assessment period, the cosmetic outcome was rated as improved by 89.0% of patients and by 87.6% of their partners (Figure 4c,d).

**FIGURE 5:**
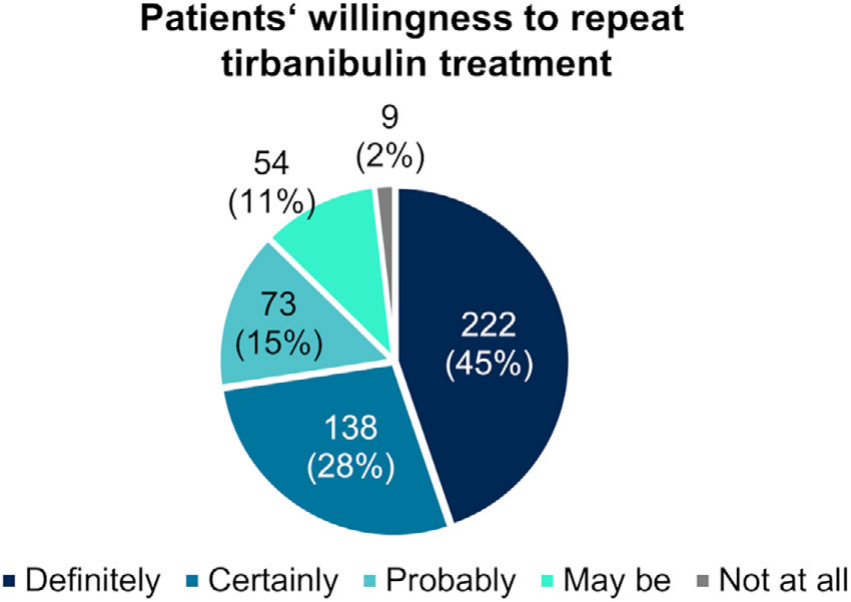
Patients’ willingness to repeat tirbanibulin treatment (n = 496) at visit 3 (approximately day 57). Due to rounding, percentages may add up to more than 100%.

Regarding patients’ future treatment preferences, almost all patients (99%) would consider using tirbanibulin again for the treatment of AK (45% “definitely,” 28% “certainly,” 15% “probably,” 11% “maybe”) (Figure 5).

## DISCUSSION

This NIS, with its large and multicentric cohort of nearly 550 patients, provided additional insightful real‐world evidence on the effectiveness, safety and PROs of topical tirbanibulin 1% for AK in Germany. Results demonstrated high effectiveness of tirbanibulin in lesion reduction, which was reflected in patients’ and physicians’ satisfaction with the treatment and the large number of patients considering tirbanibulin for future treatment.

There are a number of treatment options available for the management of AKs, including topical treatments that offer a simple and convenient approach for patients.^13^ However, most topical therapies are associated with long treatment durations (diclofenac for 60–90 days, 5‐fluorouracil for 4–12 weeks and imiquimod for two 4‐week cycles).^7^ In contrast, tirbanibulin 1% ointment requires one daily application for five consecutive days.^3^, ^18^ Consistently, the short treatment duration may have contributed to the high adherence rate of more than 90% for the 5‐day treatment regimen. This was also demonstrated in an earlier real‐world study of tirbanibulin, in which nearly 90% of patients rated their compliance with tirbanibulin as excellent or good.^17^ This in turn may contribute to the effective lesion clearance observed in this study (reduction of the total number of lesions by 70% after a mean of 72.4 days and 73% at visit 4 compared to baseline). The clearance rates of this study were generally comparable to the efficacy reported in the phase II and phase III trials, as well as another real‐life study.^3^, ^14^, ^17^

Notably, no new skin tumors in the treatment area were observed and reported AEs and LSRs are consistent with the published safety profile, further supporting the safety and good tolerability of tirbanibulin.^3^, ^14^, ^19^ The mechanism of action of the anti‐proliferative agent tirbanibulin is based on the inhibition of tubulin polymerization, which ultimately leads to apoptotic cell death.^22^, ^23^ In particular, the reversible nature of tirbanibulin binding may explain the predominantly mild or moderate LSRs observed in clinical trials. This can be beneficial for patient compliance and patients’ willingness to continue or repeat these treatment options.

To complement clinical data, PROs provide a patient‐centered perspective on treatment benefits. The results of the PROs demonstrated that the majority of patients were satisfied with the treatment. In particular, a large proportion of patients and their partners rated the cosmetic outcome as improved. Accordingly, almost all patients (99%) would consider repeating treatment with tirbanibulin indicating a high level of acceptance of the novel treatment. These results are consistent with those of the non‐interventional PROAK study, which was conducted in the US. This study also assessed PROs and documented high levels of treatment satisfaction and thus, a high likelihood of both patients and physicians considering tirbanibulin treatment in the future.^20^

As this study was only conducted in Germany, the study population may not be representative of other countries. The current study is limited by its non‐interventional design and the lack of a direct comparison group. Furthermore, it cannot be excluded that the inclusion of patients in the study may have been biased due to their willingness to participate in research studies. However, the variables measured (e.g., lesion counts, LSRs and PROs) are part of the routine clinical practice when treating AKs in Germany. The non‐interventional design allowed for the inclusion of a wide range of visit windows, resulting in the collection of data at different time points for visits 2–4. This may have introduced some bias into the comparability of the results. Moreover, the number of patients returning for visits decreased over the course of the study, which may be due to systematic bias related to treatment success or failure. Nevertheless, participation was still high throughout the study.

In conclusion, the results of this study confirm the effectiveness and the safety profile of tirbanibulin under real‐world conditions. In addition, these data highlight the benefits of tirbanibulin from a patient perspective and demonstrate that this topical treatment is highly valued by patients with AK. Looking ahead, current and future large‐scale clinical trials aim to further expand the evidence base for tirbanibulin. These include a three‐year comparative safety study with diclofenac,^24^ research of tirbanibulin application in immunocompromised populations, as well as a comprehensive European evaluation focusing on the safety and efficacy of tirbanibulin in the treatment of larger areas.^25^

## CONFLICT OF INTEREST STATEMENT

M.V.H. has been a member of advisory boards of Almirall, Sanofi, Merck Sharp & Dohme, Bristol‐Myers Squibb, Novartis, Immunocore, Pierre Fabre, and received speaker's honoraria from Galderma and Biofrontera. I.H. has served as consultant and/or speaker for AbbVie, Almirall, Galderma, Janssen, Leo Pharma, Novartis, Pfizer, Regeneron, Sanofi, and UCB Pharma. A.K. and A.M. are employees of Almirall Hermal GmbH. C.B. reports honoraria for presentations on conferences from Almirall, Bristol Myers Squibb, Novartis, Merck Sharp & Dohme, Leo Pharma, and Regeneron; participation on a data safety monitoring board or advisory board for Delcath, InflaRx, Miltenyi, Bristol Myers Squibb, Novartis, Merck Sharp & Dohme, Almirall, Pierre Fabre, Sanofi, Regeneron, and Immunocore.

## References

[1] Marques E , Chen T . Actinic Keratosis, Treasure Island (FL): StatPearls Publishing, Avalaible from: https://www.ncbi.nlm.nih.gov/books/NBK557401/ (2023) (Last accessed April 8, 2025). 32491333

[2] Richard MA , Amici JM , Basset‐Seguin N , et al. Management of actinic keratosis at specific body sites in patients at high risk of carcinoma lesions: expert consensus from the AKTeam of expert clinicians. J Eur Acad Dermatol Venereol. 2018;32:339‐346. 29235161 10.1111/jdv.14753

[3] Blauvelt A , Kempers S , Lain E , et al. Phase 3 Trials of Tirbanibulin Ointment for Actinic Keratosis. N Engl J Med. 2021;384:512‐520. 33567191 10.1056/NEJMoa2024040

[4] Cockerell CJ , Wharton JR . New histopathological classification of actinic keratosis (incipient intraepidermal squamous cell carcinoma). J Drugs Dermatol. 2005;4:462‐467. 16004019

[5] Yantsos VA , Conrad N , Zabawski E , et al. Incipient intraepidermal cutaneous squamous cell carcinoma: a proposal for reclassifying and grading solar (actinic) keratoses. Semin Cutan Med Surg. 1999;18:3‐14. 10188837 10.1016/s1085-5629(99)80003-0

[6] Fernandez Figueras MT . From actinic keratosis to squamous cell carcinoma: pathophysiology revisited. J Eur Acad Dermatol Venereol. 2017;31(Suppl 2):5‐7. 10.1111/jdv.14151 28263020

[7] Heppt MV , Leiter U , Steeb T , et al. S3 guideline for actinic keratosis and cutaneous squamous cell carcinoma ‐ short version, part 1: diagnosis, interventions for actinic keratoses, care structures and quality‐of‐care indicators. J Dtsch Dermatol Ges. 2020;18:275‐294. 10.1111/ddg.14048 32130773

[8] Fernandez‐Figueras, M. T. , Carrato, C. , Saenz, X . et al. Actinic keratosis with atypical basal cells (AK I) is the most common lesion associated with invasive squamous cell carcinoma of the skin. J Eur Acad Dermatol Venereol. 2015;29:991‐997. 25428612 10.1111/jdv.12848

[9] Heppt MV , Leiter U , Steeb T , et al. [S3‐Leitlinie "Aktinische Keratose und Plattenepithelkarzinom der Haut" – Update 2023, Teil 1: Therapie der aktinischen Keratose, Morbus Bowen, Cheilitis actinica, berufsbedingte Erkrankung und Versorgungsstrukturen: S3 guideline “actinic keratosis and cutaneous squamous cell carcinoma”. J Dtsch Dermatol Ges. 2023;21:1249‐1262. 10.1111/ddg.15231_g 37845050

[10] Kandolf L , Peris K , Malvehy J . et al. European consensus‐based interdisciplinary guideline for diagnosis, treatment and prevention of actinic keratoses, epithelial UV‐induced dysplasia and field cancerization on behalf of European Association of Dermato‐Oncology, European Dermatology Forum, European Academy of Dermatology and Venereology and Union of Medical Specialists (Union Européenne des Médecins Spécialistes). J Eur Acad Dermatol Venereol. 2024;38:1024‐1047. 38451047 10.1111/jdv.19897

[11] Freeman M , Vinciullo C , Francis D , et al. A comparison of photodynamic therapy using topical methyl aminolevulinate (Metvix) with single cycle cryotherapy in patients with actinic keratosis: a prospective, randomized study. J Dermatolog Treat. 2003;14:99‐106. 12775317 10.1080/09546630310012118

[12] Markham, A . Duggan, S . Tirbanibulin: First Approval. Drugs. 2021;81:509‐513. 33713299 10.1007/s40265-021-01479-0

[13] Pellacani G , Schlesinger T , Bhatia N , et al. Efficacy and safety of tirbanibulin 1% ointment in actinic keratoses: Data from two phase‐III trials and the real‐life clinical practice presented at the European Academy of Dermatology and Venereology Congress 2022. J Eur Acad Dermatol Venereol. 2024;38(Suppl 1):3‐15. 10.1111/jdv.19636 38116638

[14] Kempers S , DuBois J , Forman S , et al. Tirbanibulin Ointment 1% as a Novel Treatment for Actinic Keratosis: Phase 1 and 2 Results. J Drugs Dermatol. 2020;19:1093‐1100. 33196758 10.36849/JDD.2020.5576

[15] Bhatia N , Lain E , Jarell A , et al. Safety and tolerability of tirbanibulin ointment 1% treatment on 100 cm2 of the face or scalp in patients with actinic keratosis: A phase 3 study. JAAD International. 2024;17:6‐14. 39268198 10.1016/j.jdin.2024.07.001 PMC11387381

[16] DuBois J , Jones T M , Lee MS , et al. Pharmacokinetics, Safety, and Tolerability of a Single 5‐Day Treatment of Tirbanibulin Ointment 1% in 100 cm2: A Phase 1 Maximal‐Use Trial in Patients with Actinic Keratosis. Clin Pharmacol Drug Dev. 2024;13:208‐218. 38185925 10.1002/cpdd.1368

[17] Li Pomi F , Vaccaro M , Pallio G , et al. Tirbanibulin 1% Ointment for Actinic Keratosis: Results from a Real‐Life Study. Medicina (Kaunas). 2024;60:225. 38399512 10.3390/medicina60020225 PMC10890708

[18] Kirchberger MC , Gfesser M , Erdmann M , et al. Tirbanibulin 1% Ointment Significantly Reduces the Actinic Keratosis Area and Severity Index in Patients with Actinic Keratosis: Results from a Real‐World Study. J Clin Med. 2023;12:4837. 37510952 10.3390/jcm12144837 PMC10381110

[19] Nazzaro G , Carugno A , Bortoluzzi P , et al. Efficacy and tolerability of tirbanibulin 1% ointment in the treatment of cancerization field: a real‐life Italian multicenter observational study of 250 patients. Int J Dermatol. 2024;63:1566‐1574. 38605473 10.1111/ijd.17168

[20] Schlesinger T , Kircik L , Lebwohl M , et al. Patient‐ and Clinician‐Reported Outcomes for Tirbanibulin in Actinic Keratosis in Clinical Practice Across the United States (PROAK). J Drugs Dermatol. 2024;23:338‐346. 38709702 10.36849/JDD.8264

[21] Kose O , Koc E , Erbil AH , et al. Comparison of the efficacy and tolerability of 3% diclofenac sodium gel and 5% imiquimod cream in the treatment of actinic keratosis. J Dermatolog Treat. 2008;19:159‐163. 18569272 10.1080/09546630701818870

[22] Dao DD , Sahni VN , Sahni DR , et al. 1% Tirbanibulin Ointment for the Treatment of Actinic Keratoses. Ann Pharmacother. 2022;56:494‐500. 34301153 10.1177/10600280211031329 PMC8899810

[23] Gilchrest BA . Tirbanibulin: A New Topical Therapy for Actinic Keratoses With a Novel Mechanism of Action and Improved Ease of Use. Clin Pharmacol Drug Dev. 2021;10:1126‐1129. 34612001 10.1002/cpdd.1024

[24] A Study of Tirbanibulin Ointment and Diclofenac Sodium Gel for the Treatment of Adult Participants With Actinic Keratosis on the Face or Scalp (AKtive) , ClinicalTrials.gov. Avalaible from: https://clinicaltrials.gov/study/NCT05387525?intr=Tirbanibulin&rank=10&page=1&limit=10 (2025) (Last accessed April 8, 2025).

[25] A Study to Evaluate the Efficacy and Safety of Tirbanibulin Ointment in Adult Participants With Actinic Keratosis (TirbAKare) , ClinicalTrial.giv. Avalaible from: https://clinicaltrials.gov/study/NCT06135415?intr=Tirbanibulin&rank=6&page=1&limit=10 (2025) (Last accessed April 8, 2025).

